# Novel insights from structural analysis of lentiviral and gammaretroviral reverse transcriptases in complex with RNA/DNA hybrids

**DOI:** 10.1186/1742-4690-10-S1-P46

**Published:** 2013-09-19

**Authors:** Stuart Le Grice, Mikalai Lapkouski, Lan Tian, Jennifer Miller, Elzbieta Nowak, Wojciech Potrzebowski, Peter Konarev, Jason Rausch, Marion Bona, Dmitri Svergun, Janusz Bujnicki, Marcin Nowotny, Wei Yang

**Affiliations:** 1 HIV DRP, National Cancer Institute, Frederick, MD, USA; 2 NIDDK, National Institutes of Health, Bethesda, MD, USA; 3 International Institute of Molecular & Cell Biology, Warsaw, Poland; 4 European Molecular Biology Laboratory, Hamburg, Germany

## 

Structures of HIV-1 reverse transcriptase (RT) have been reported in several forms, but only one contains an RNA/DNA hybrid, the conformation of which has been controversial. We have been successful in obtaining three structures of HIV-1 RT complexed with a non-nucleoside RT inhibitor (NNRTI) and an RNA/DNA hybrid [1]. In the presence of an NNRTI, our RNA/DNA structure differs from all prior nucleic acid bound to RT including the previously-reported RNA/DNA hybrid derived froom the polypurine tract. The enzyme structure observed in our cocrystals also differs from all previous RT-DNA complexes. As a result, the hybrid has ready access to the ribonuclease H (RNase H) active site. These observations collectively reinforce previous proposals that an RT-nucleic acid complex may be required to adopt independent structural states competent for DNA synthesis and the other for RNA degradation. RT mutations that confer drug resistance but are distant from the inhibitor-binding sites map to the unique RT-hybrid interface that undergoes conformational changes between two catalytic states. Structural features of the nucleoprotein complex, including drug resistance mutations, have been verified by site-directed mutagenesis, and will be presented.

Although the single-subunit RT of Moloney murine leukemia virus (Mo-MLV) has been extensively characterized biochemically, structural information is lacking that describes the substrate binding mechanism for this RT species. We also present data on the first crystal structure of a complex between an RNA/DNA hybrid and the 72 kDa single-subunit RT from the related xenotropic murine leukemia virus-related virus (XMRV) [2]. A comparison of this structure with its HIV-1 counterpart shows that substrate binding around the DNA polymerase active site is conserved but differs between the two enzymes in their thumb and connection subdomains. Small-angle X-ray scattering (SAXS) was used to model full-length XMRV RT, demonstrating its flexible RNase H domain becomes ordered in the presence of substrate, a key difference between monomeric and dimeric RTs.
